# Combined Systemic Immune-inflammatory Index (SII) and Geriatric Nutritional Risk Index (GNRI) predict survival in elderly patients with hip fractures: a retrospective study

**DOI:** 10.1186/s13018-024-04585-3

**Published:** 2024-02-06

**Authors:** Ling Zhou, Chao Huang, Xianjie Zhu, Zhenhua Ma

**Affiliations:** 1 Graduate School of Dalian Medical University, Dalian, China; 2 Department of Orthopaedic Surgery, Qingdao Municipal Hospital, Qingdao, China; 3 Department of Orthopaedic Surgery, People’s Hospital of Rizhao, Rizhao, China

**Keywords:** Elderly, Hip fracture, Surgery, Mortality, Geriatric Nutritional Risk Index, Systemic Immune-inflammatory Index

## Abstract

**Purpose:**

The Systemic Immune-inflammatory Index (SII) and Geriatric Nutritional Risk Index (GNRI) have undergone comprehensive examination and validation in forecasting the outcomes of diverse medical conditions. Nevertheless, the correlation between the combined use of GNRI and SII metrics and hip fractures has yet to be elucidated. This study aimed to determine whether the amalgamation of SII and GNRI scores constitutes an independent prognostic factor for elderly patients with hip fractures.

**Methods:**

We conducted a retrospective analysis of elderly patients admitted to our facility with hip fractures, encompassing both femoral neck and intertrochanteric fractures. Demographic information, experimental parameters, and postoperative complications were systematically recorded. The Geriatric Nutritional Risk Index (GNRI) and Systemic Immunoinflammatory Index (SII) were meticulously computed. Receiver operating characteristic (ROC) curves were generated, and optimal cutoff values for each parameter were determined. Subsequently, a multivariate Cox regression analysis was employed to assess the predictive utility of the SII–GNRI score in relation to 1-year postoperative mortality among elderly patients with hip fractures.

**Results:**

In a study involving 597 patients, 90 of whom experienced mortality within 1 year, it was observed that the SII-GNRI score in the group of patients who passed away was significantly higher compared to the group that survived. Following a multifactorial adjustment, it was established that a high SII–GNRI score served as an independent predictor of 1-year all-cause mortality in older patients with hip fractures. In addition to the SII–GNRI score, factors such as length of hospital stay, CCI > 2, and blood transfusion were also identified as independent risk factors for survival. Notably, the incidence of postoperative complications in patients with high SII–GNRI scores was significantly greater than in patients with low scores.

**Conclusion:**

The SII–GNRI score proves valuable in predicting the 1-year survival rate for elderly patients with hip fractures who have undergone surgery.

## Introduction

As the global population ages, fragility fractures, primarily due to osteoporosis, have become a common concern among the elderly worldwide, primarily resulting from indirect external forces, with femoral neck fractures and intertrochanteric fractures being the most prevalent [1, 2]. Projections indicate that, by 2050, worldwide hip fractures are anticipated to range from 6.26 million to a maximum of 21.3 million [3, 4], with approximately 50% occurring in Asia [5]. Hip fractures significantly impact the physical and mental well-being of elderly patients, and complications resulting from underlying diseases can worsen their condition. The mortality rate during the first year following a fracture varies from 8% to 36%, and a considerable number of survivors may necessitate long-term home care, which results in a substantial and rapidly increasing financial burden on patients, their families, and the healthcare system [2, 6–9]. Surgical intervention is often considered the optimal choice for restoring pre-injury daily life capabilities and mitigating complications associated with extended bed rest, such as pressure ulcers, pulmonary infections, deep vein thrombosis in the lower limbs, and muscle atrophy [10]. Nevertheless, preoperative factors such as hemoglobin levels, nutritional status, immune health, loss of skeletal muscle mass, and inflammatory responses can elevate the risk of postoperative adverse outcomes, leading to extended hospital stays, diminished quality of life, and increased mortality [11–13]. It is imperative to identify high-risk patients and modify risk factors while enhancing perioperative management in order to reduce the incidence of postoperative complications and mortality. Prior research has indicated predictive models for hip fracture complications, including the ACS NSQIP Surgical Risk Calculator [14] and the Nottingham Hip Fracture Score [15]. However, our study employs an approach that integrates inflammatory and nutritional status to offer a more comprehensive prognosis assessment and prediction for elderly patients with hip fractures.

Insufficient caloric intake, metabolic changes, and increased nutrient loss in elderly patients have historically resulted in malnutrition, a condition universally recognized for its deleterious effects on various physiological systems, culminating in heightened postoperative complications and mortality rates [16]. Simultaneously, the corpus of research has consistently demonstrated the profound association between immunoinflammatory cells and the less favorable prognosis and postoperative complications frequently observed in hip fracture patients. Notable parameters, including the neutrophil–lymphocyte ratio (NLR) [17], platelet–lymphocyte ratio (PLR) [18], and monocyte–lymphocyte ratio (MLR) [19], have been the focus of extensive investigation. Elderly patients, characterized by their diminished physiological reserves and the insufficiency of anti-inflammatory mediators to maintain homeostasis, tend to experience a heightened release of inflammatory cytokines following injury. This susceptibility to an exacerbated inflammatory response significantly contributes to the less favorable prognosis for these patients [20].

The Systemic Immunoinflammatory Index (SII), a contemporary addition to the repertoire of inflammatory metrics, is computed based on the peripheral blood composition of neutrophils, platelets, and lymphocytes, providing insight into distinct inflammatory and immune pathways inherent to the body. Notably, when compared with the platelet-to-lymphocyte ratio and neutrophil-to-lymphocyte ratio, the SII demonstrates superior stability [21]. Research by Wang et al. has demonstrated a pronounced and statistically significant link between SII and all-cause postoperative mortality within the demographic of elderly hip fracture patients [22].

The Geriatric Nutritional Risk Index (GNRI), as utilized in assessing the nutritional status of elderly patients and projecting clinical outcomes [23], has historically relied upon the straightforward measurement of weight, height, and serum albumin levels. When juxtaposed with other nutritional assessment instruments, such as the Mini Nutritional Assessment (MNA) and the Subjective Global Assessment (SGA), the GNRI stands as a notably more objective and accurate tool for identifying malnutrition. Numerous research endeavors have substantiated the predictive capacity of the Systemic Immune Inflammation Index (SII) and the Geriatric Nutritional Risk Index (GNRI) in foretelling the outcomes of various malignancies, including non-small cell lung cancer [24, 25], hepatocellular carcinoma [25, 26] and hip fractures [22, 27]. Nonetheless, the integration of SII with GNRI to evaluate the prognosis of elderly patients following hip fracture surgery remains a relatively unexplored terrain. Within the confines of this retrospective investigation, we undertook the assessment of the SII–GNRI score upon admission for elderly patients recovering from hip fractures post-surgery. This assessment was undertaken to discern predictive survival patterns and to provide informed guidance for perioperative management.

## Methods

### Study design and patients

In this retrospective cohort study, 597 patients with hip fractures (including femoral neck and intertrochanteric femur fractures) were enrolled. The study was conducted at Qingdao Municipal Hospital from January 2016 to December 2020. The inclusion criteria for this study were as follows: (1) participants aged 60 years or older; (2) individuals diagnosed with hip fractures, including femoral neck fractures and femoral intertrochanteric fractures; (3) fractures caused by a fall from a height no greater than standing height; and (4) fresh fractures that occurred within two weeks. Patients meeting any of the following criteria were excluded from the study: high-energy fractures, pathological fractures, a history of cancer, preoperative hemiplegia, incomplete data, and lack of follow-up. This study obtained approval from the hospital's ethics review committee, as the data utilized were retrospective and anonymized. Patient consent was waived, and the study adhered to the principles outlined in the Helsinki Declaration.

### Data collection

Patient characteristics, including sex, age, details about the fracture (cause, type, and location), the presence of essential hypertension, type 2 diabetes, and heart diseases such as coronary heart disease, myocardial infarction, and heart failure, as well as preoperative anemia, pneumonia, liver disease, and Cerebral disease such as Alzheimer’s disease, Parkinson's disease, and a history of cerebral infarction, were collected. Additionally, comorbidities were assessed using the Charlson Comorbidities Index (CCI), and the American Society of Anesthesiologists physical score (ASA) was determined. The duration of the hospital stay before surgery was also recorded. Intraoperative data included the type of surgical procedure performed, the extent of blood loss, and instances of blood transfusion. Routine hematological tests, covering parameters such as hemoglobin, albumin, neutrophil counts, lymphocyte counts, and platelet counts, are conducted as a standard procedure for all patients either in the emergency department or upon admission. The Nutritional Risk Index (GNRI) and Systemic Immunoinflammatory Index (SII) were computed for each patient. GNRI was calculated using the formula: GNRI = [14.89 × serum albumin (g/L)] + [41.7 × present/ideal body weight (kg)], with ideal body weight defined as 22 times the patient's height (m) squared [28]. On the other hand, SII was determined using the formula: SII = platelet count × neutrophil count/lymphocyte count [29]. Postoperative complications observed during the hospital stay included pneumonia, respiratory failure, gastrointestinal bleeding, pulmonary embolism, arrhythmia, angina, myocardial infarction, heart failure, stroke, and mortality.

### Follow-up

In our study, follow-ups were primarily conducted via phone, focusing on patients' post-surgical recovery. If a patient’s death was identified, we recorded the time and cause of death. The main endpoint of the study is the all-cause mortality rate within one year of admission, with subjects who passed away within this period classified in the death group. Patients or their relatives who could not be contacted were considered as lost to follow-up and were excluded from the study.

### Statistical analysis

Continuous variables are presented as mean ± standard deviation (SD) or median (interquartile range, IQR), depending on the data distribution. Normally distributed variables were analyzed using the Independent Student’s *t*-test, while the Mann–Whitney test was applied for non-normally distributed variables. Categorical variables were expressed as frequencies (percentage), and statistical comparisons were conducted employing the *χ*2 test or Fisher precision test. Furthermore, the risk of death was analyzed using a multivariate Cox proportional hazards model after adjusting for significant variables identified in univariate analysis (*p* < 0.10). The relationship between GNRI and SII was evaluated through Spearman correlation analysis. Statistical analyses were conducted using SPSS 26 and R 4.3.1, with statistical significance set at *p* < 0.05.

## Results

### Patient characteristics

**Fig. 1:**
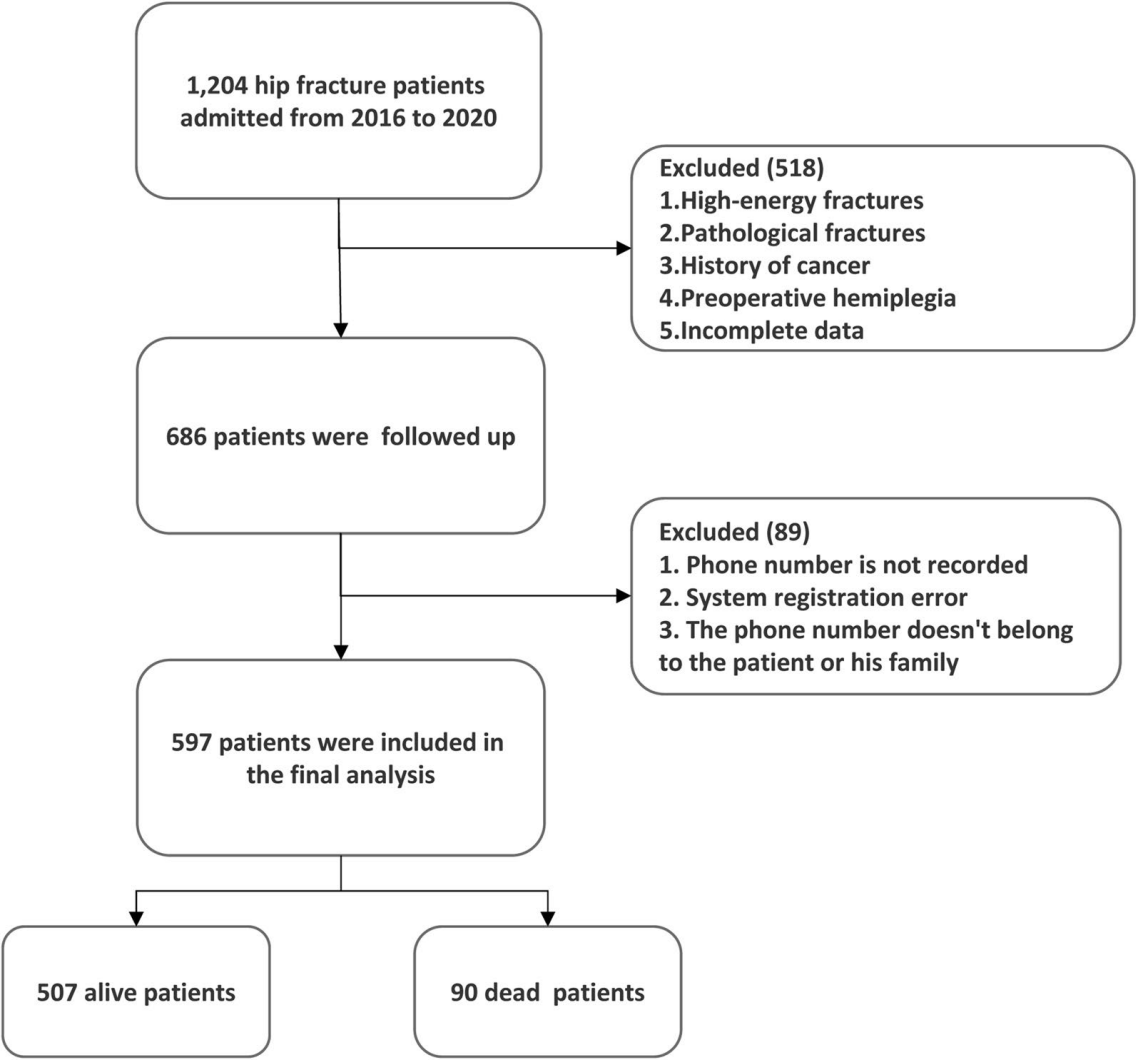
Flow chart of patients included in the study

**Fig. 2:**
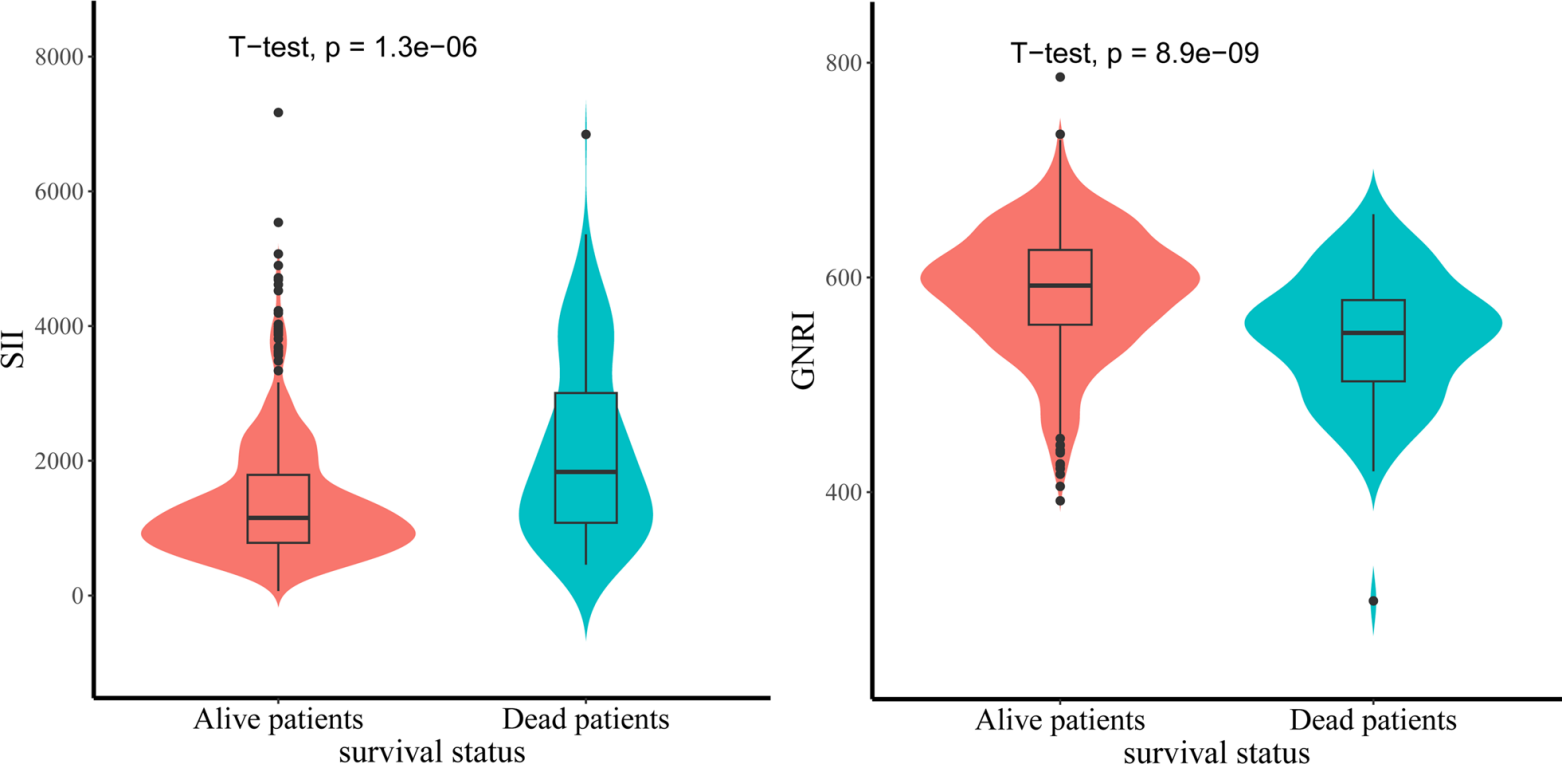
Violin plots comparing SII and GNRI between alive and dead patients. The horizontal lines within the violin represent the median; the boxes represent the interquartile range; the bars represent the 95% confidence intervals; the width of the shapes represents the density of distribution. Differences between groups were analyzed by Wilcoxon rank-sum test

A total of 1204 surgical patients in our department underwent screening, with 597 patients ultimately included in the final analysis (Table 1 and Fig. 1). The patient cohort primarily consisted of females, totaling 468 cases. Their mean age was 77.55 ± 10.90 years, with 291 patients being aged 80 years or older. Among them, 348 had a history of hypertension, 175 were diagnosed with diabetes, and 74 exhibited a CCI score of greater than 2. Additionally, 344 patients had an ASA score exceeding 3. Intertrochanteric fractures represented 41.98% of the cases, and 38.05% received red blood cell transfusions. Upon admission, the mean values for albumin, height, and weight were 35.95 ± 4.05 g/L, 162.64 ± 7.85 cm, and 63.00 ± 11.28 kg, respectively. Laboratory test results revealed platelet counts of 210.87 ± 75.45 × 10^9^/L, lymphocyte counts of 1.14 ± 0.51 × 10^9^/L, and lymphocyte percentages of 7.03 ± 2.86 × 10^9^/L. Notably, 98 patients experienced postoperative complications, and within one year, 90 cases had unfortunately passed away, resulting in a mortality rate of 14.15%. It's worth noting that the Systemic Immunoinflammatory Index (SII) of deceased patients was significantly higher than that of survivors (*P* = 0.048), while the Geriatric Nutritional Risk Index (GNRI) of deceased patients was lower than that of survivors (*P* = 0.038) (Fig. 2). Additionally, Spearman correlation analysis revealed no significant correlation between SII and GNRI (*r* = − 0.079, *P* = 0.054).

**Table 1:** Clinical characteristics of study patients

Variable	Total, *n* = 597	Survival status		*P* value
		Alive patients (507)	Dead patients (90)	
Age, years (Mean,SD)	77.55 ± 10.90	76.93 ± 11.24	81.04 ± 10.91	0.001
Age ≥ 80 years, *n*	291	236	55	0.011
Female, *n*	468	400	68	0.478
Hypertension, *n*	348	290	58	0.199
Diabetes, *n*	175	147	28	0.684
Cerebral disease, *n*	128	100	28	0.013
Heart disease, *n*	191	150	41	0.003
Charlson Comorbidities Index > 2, *n*	74	47	27	< 0.001
ASA > 2, *n*	344	276	68	< 0.001
Hip fracture type				
Intertrochanteric, *n*	257	203	54	
Neck, *n*	340	304	36	< 0.001
Length of stay, day (Mean, SD)	11.30 ± 9.24	10.28 ± 6.36	17.07 ± 17.39	< 0.001
Blood transfusion, *n*	227	169	58	< 0.001
Anemia, *n*	183	129	54	< 0.001
Albumin, × g/L (Mean, SD)	35.95 ± 4.05	36.36 ± 3.90	33.64 ± 4.12	< 0.001
NEU count, × 10^9^/L (Mean, SD)	7.03 ± 2.86	6.77 ± 2.65	8.50 ± 3.46	< 0.001
LYM count, × 10^9^/L (Mean, SD)	1.14 ± 0.51	1.14 ± 0.46	1.19 ± 0.70	0.370
PLT, × 10^9^/L (Mean, SD)	210.87 ± 75.45	205.19 ± 72.03	242.89 ± 86.014	< 0.001
Height, × CM (Mean, SD)	162.64 ± 7.85	162.61 ± 7.78	162.84 ± 8.26	0.977
Weight, × Kg (Mean, SD)	63.00 ± 11.28	63.52 ± 11.01	60.00 ± 12.30	0.023
GNRI (Mean, SD)	580.42 ± 61.41	586.93 ± 59.13	543.73 ± 61.42	< 0.001
SII (Mean, SD)	1521.34 ± 1047.00	1404.58 ± 931.38	2179.13 ± 1376.17	< 0.001
POCs, *n*	98	56	42	< 0.001
1-year death, *n*	90			

*ASA* American Society of Anesthesiologists physical score, *NEU* neutrophil, *LYM* lymphocyte, *PLT* Platelet, *GNRI* Geriatric Nutritional Risk Index, *SII* Systemic Immune-inflammatory Index, *POCs* postoperative complications

*Heart disease* coronary heart disease, myocardial infarction and heart failure; *Cerebral disease* Alzheimer's disease, Parkinson's disease and a history of cerebral infarction

### The optimal GNRI and SII cutoff were calculated by the ROC curve

**Fig. 3:**
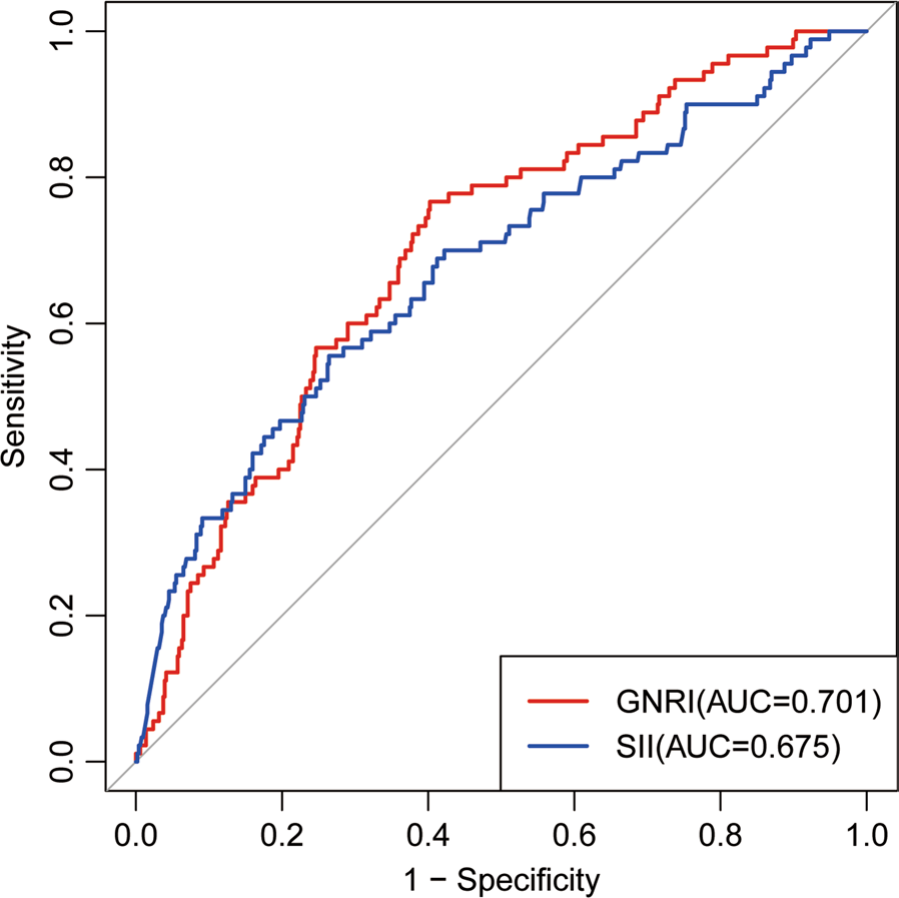
Receiver operating characteristic (ROC) curves of SII and GNRI for predicting 1-year survival. Data presented as area under the curve (AUC) with 95% confidence interval (CI)

Based on the ROC curve (Fig. 3), the optimal cutoff for GNRI was determined to be 579.39, with an AUC of 0.701, sensitivity of 76.7%, and specificity of 59.8%. Similarly, the best cutoff for SII was found to be 1621.92, resulting in an AUC of 0.675, a sensitivity of 55.6%, and a specificity of 73.6%. We conducted an assessment of the influence of various preoperative factors on SII and GNRI. In the univariate analysis, factors such as age exceeding 80 years (OR, 1.6; 95% CI 1.12–2.28, *P* = 0.01), intertrochanteric fractures, the presence of diabetes, and admission with low albumin levels were found to be associated with an increased risk of SII levels equal to or exceeding 1621.92 (Table 2). Furthermore, age greater than 80 years and intertrochanteric fractures were linked to an elevated risk of GNRI levels falling below 579.39. With regard to the optimal SII and GNRI thresholds, we classified the patients into three groups:

**Table 2:** Preoperative factors associated with the SII and GNRI

Characteristic	SII		GNRI	
	OR (95% CI)	*P* Value	OR (95% CI)	*P* Value
Age (< 80 vs. ≥ 80 years)	1.6 (1.12–2.28)	0.01	1.919 (1.385–2.659)	0.015
Gender (male vs. female)	0.90 (0.59–1.36)	0.62	1.8 (0.928–2.053)	0.112
CCI (≤ 2 vs. > 2)	1.426 (0.852–2.385)	0.177	1.294 (0.795–2.016)	0.30
Hypertension	0.815 (0.569–1.166)	0.263	0.82 (0.727–1.449)	0.883
Diabetes	0.637 (0.422–0.961)	0.032	0.999 (0.701–1.571)	0.235
Cerebral disease	1.191 (0.778–1.822)	0.421	1.061 (0.717–1.571)	0.766
Heart disease	1.059 (0.725–1.547)	0.768	1.026 (0.717–1.571)	0.766
Anemia	0.965 (0.668–1.361)	0.794	0.903 (0.654–1.246)	0.534
Fracture type (Intertrochanteric vs. neck)	0.697 (0.488–0.997)	0.048	0.671 (0.484–0.93)	0.016
ASA (≤ 2 vs. > 2)	1.147 (0.799–1.646)	0.458	1.171 (0.845–1.623)	0.344
Albumin (< 35 vs. ≥ 35 g/L)	0.952 (0.911–0.994)	0.027	–	–

*ASA* American Society of Anesthesiologists physical score, *CCI* Charlson Comorbidities Index, *Heart disease* coronary heart disease, myocardial infarction and heart failure; *Cerebral disease* Alzheimer's disease, Parkinson's disease and a history of cerebral infarction

A score of 1 for individuals with low SII (SII < 1621.92) and high GNRI (GNRI > 579.39).

A score of 2 for individuals with high SII (SII ≥ 1621.92) and high GNRI (GNRI > 579.39), or low SII (SII < 1621.92) and low GNRI (GNRI ≤ 579.39).

A score of 3 for individuals with high SII (SII ≥ 1621.92) and low GNRI (GNRI ≤ 579.39).

The combined SII and GNRI scores for deceased patients were markedly higher compared to those of the survivors (Table 3).

**Table 3:** Relationship between survival and complications and SII-GRNI score

	SII–GNRI score			*P* value
	1	2	3	
No-POCs, n	224	217	58	0.001
POCs, n	19	47	32	
Alive patients, n	233	219	55	0.001
Dead patients, n	10	45	35	

*POCs* postoperative complications

### Clinical variables predicting one-year mortality

**Fig. 4:**
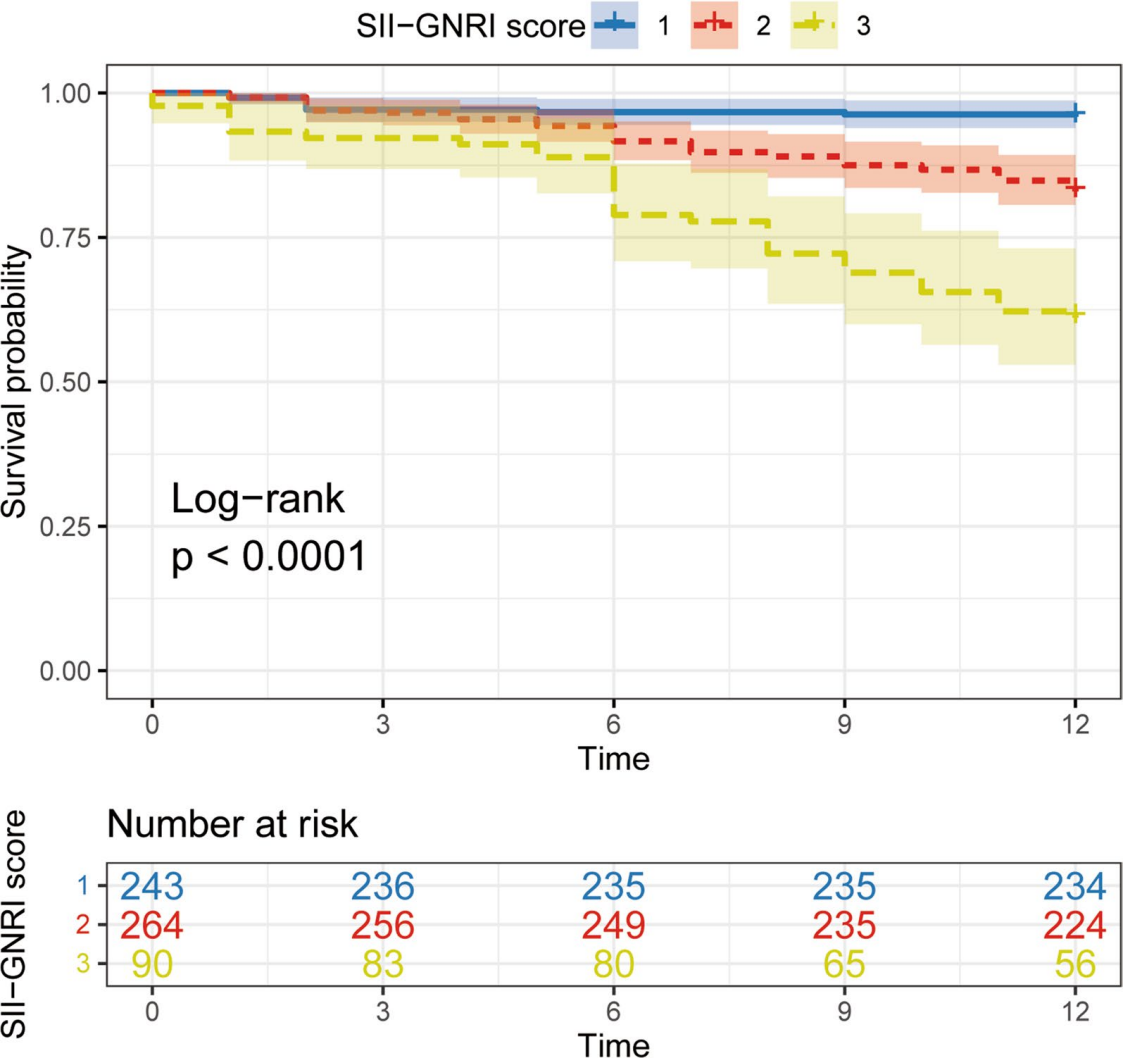
Kaplan–Meier survival analyses of elderly hip fracture patients according to SII–GNRI score. Hazard ratio (HR) was calculated using a Cox regression analysis, and p value was estimated using the log-rank test, and the shaded areas indicate 95% confidence interval (CI). The numbers shown below the Kaplan–Meier survival curves are the number of patients at risk at the specified month for each group

Table 4 displays the relationship between clinical variables and 1-year mortality. In the COX regression unifactor analysis, several factors, such as older age (≥ 80 years), CCI > 2, fracture site, heart disease, brain disease, anemia, length of hospital stay, blood transfusion, ASA > 2, GNRI ≤ 579.39, SII ≥ 1621.92, low albumin levels, low body weight, and high SII–GNRI score (2–3), were found to be significantly associated with an increased 1-year mortality. As SII–GNRI incorporates albumin, weight, and height, it has been excluded from the multivariate Cox proportional risk model. In the multivariate analysis, the inclusion of significant factors revealed that the SII–GNRI score = 3 (HR 8.47, 95% CI 4.10–17.49), length of hospital stay (HR 1.02, 95% CI 1.00–1.03), CCI > 2 (HR 2.10, 95% CI 1.24–3.53), blood transfusion (HR 2.20, 95% CI 1.40–3.46), and SII–GNRI score = 2 (HR 3.68, 95% CI 1.83–7.40) emerged as risk factors for 1-year mortality in elderly patients with hip fractures (*P* < 0.05).These findings were further supported by the log-rank test of Kaplan–Meier curve analysis, which demonstrated that patients with a higher SII combined GNRI score had a higher mortality rate (Fig. 4), indicating that elderly patients with high SII and low GNRI had a poorer prognosis.

**Table 4:** The univariate and multivariate Cox regression analysis of factors associated with 1-year all-cause mortalitylight"/>

Variable	1–year mortality			
	Univariate		Multivariate	
	HR (95% CI)	*P* value	HR (95% CI)	*P* value
Age(years)				
< 80	1.0 (Reference)			
≥ 80	1.69 (1.11–2.59)	0.015	0.76 (0.58–1.48)	0.757
Gender				
Male	1.0 (Reference)			
Female	0.86 (0.53–1.39)	0.543	–	–
Charlson Comorbidities Index				
≤ 2	1.0 (Reference)			
> 2	3.51 (2.23–5.51)	0.000	2.10 (1.24–3.53)	0.005
Hypertension	1.31 (0.85–2.02)	0.216	–	–
Diabetes	1.10 (0.71–1.72)	0.673	–	–
Cerebral disease	1.75 (1.12–2.74)	0.014	1.05 (0.65–1.71)	0.844
Heart disease	2.86 (1.23–2.82)	0.003	1.33 (0.85–2.09)	0.216
Anemia	3.74 (2.45–5.70)	< 0.001	1.39 (0.90–2.14)	0.135
Fracture type				
Intertrochanteric	1.0 (Reference)			
Neck	0.48 (0.32–0.73)	0.001	0.74 (0.47–1.16)	0.186
American Society of Anesthesiologists physical score				
≤ 2	1.0 (Reference)			
> 2	2.39 (1.48–3.87)	< 0.001	1.59 (0.94–2.71)	0.085
Albumin (per 1 g/L increase)	0.87 (0.88–0.91)	< 0.001	–	–
Height(per 1 cm increase)	1.00 (0.98–1.03)	0.886	–	–
Weight(per 1 kg increase)	0.97 (0.96–0.99)	0.006	–	–
SII	2.69 (1.78–4.07)	< 0.001	–	–
GNRI	4.27 (2.62–6.96)	< 0.001	–	–
SII–GNRI score 1	1.0 (Reference)			
SII–GNRI score 2	4.35 (2.19–8.63)	< 0.001	3.68 (1.83–7.40)	< 0.001
SII–GNRI score 3	11.27 (5.58–22.77)	< 0.001	8.47 (4.10–17.49)	< 0.001
length of stay (per 1 day increase)	1.03 (1.02–1.04)	< 0.001	1.02 (1.00–1.03)	0.011
Blood transfusion (yes vs. no)	3.26 (2.11–5.01)	< 0.001	2.20 (1.40–3.46)	0.001

*GNRI* Geriatric Nutritional Risk Index, *SII* Systemic Immune-inflammatory Index, *Heart disease* coronary heart disease, myocardial infarction and heart failure; *Cerebral disease* Alzheimer's disease, Parkinson's disease and a history of cerebral infarction

### Predicting postoperative complications

In this study, 98 patients experienced various postoperative complications (POCs), including pulmonary infections (48 cases), deep vein thrombosis (23 cases), postoperative delirium (26 cases), acute cerebral infarction (10 cases), acute myocardial infarction (3 cases), urinary tract infections (10 cases), wound infections (6 cases), and pulmonary thromboembolism (4 cases). Our investigation revealed a significantly higher incidence of POCs among patients with high SII–GNRI scores when compared to those with lower scores (Table 3; *p* = 0.001).

## Discussion

In the field of orthopedics, inflammatory systemic biomarkers such as the Systemic Immune-inflammatory Index (SII) and geriatric nutritional indicators like the Geriatric Nutritional Risk Index (GNRI) have been employed to predict survival rates [30, 31] and postoperative complication rates. Nonetheless, this study marks the pioneering use of the SII–GNRI score to forecast one-year mortality subsequent to surgical intervention for hip fractures, encompassing both femoral neck and intertrochanteric femoral fractures. Our investigation revealed that elevated preoperative SII–GNRI scores were indicative of poorer survival, a correlation that retained statistical significance even after accounting for potential confounding factors. Additionally, patients with elevated SII–GNRI scores exhibited a heightened incidence of postoperative complications. Consequently, the preoperative SII–GNRI score can be swiftly employed to identify high-risk patients with adverse health conditions and an unfavorable prognosis, all at a relatively modest cost.

Prior research has consistently demonstrated that an elevated neutrophil-to-lymphocyte ratio (NLR) represents an independent risk factor for postoperative myocardial injury, in-hospital mortality, and one-year mortality following hip fractures [17, 32]. Furthermore, a high platelet count has been recognized as a risk factor for the development of pressure ulcers subsequent to hip fractures [33], and an elevated platelet-to-lymphocyte ratio (PLR) has been linked to an increased rate of all-cause mortality, particularly among older patients [34]. As a marker of cellular immune inflammation, SII amalgamates neutrophils, platelets, and lymphocytes, offering an objective reflection of systemic inflammation. In contrast to conventional blood-derived biomarkers such as the neutrophil-to-lymphocyte ratio (NLR) and platelet-to-lymphocyte ratio (PLR), SII can more accurately depict the inflammatory and immune status of patients, rendering it a more reliable predictor [35–38].

Between 18.7% and 45.7% of elderly patients suffering from hip fractures present with malnutrition upon admission, a condition exacerbated by a pronounced catabolic state resulting from factors such as reduced dietary intake, blood loss, and post-traumatic inflammation [39, 40], ultimately increasing the occurrence of complications. Notably, Li and colleagues [41] have established that serum albumin levels serve as an independent predictor of mortality subsequent to hip fractures. Timely identification of this condition and the provision of nutritional support have the potential to effectively reduce postoperative complications and mortality rates [42, 43]. The Geriatric Nutritional Risk Index (GNRI), which correlates with Body Mass Index (BMI) and serum albumin levels, has garnered recognition as a significant prognostic indicator across various medical conditions. These indicators are not only readily attainable but also cost-effective [44]. Within the context of systemic inflammatory responses, pro-inflammatory cytokines and growth factors exert catabolic effects that contribute to muscle breakdown. This may further lead to localized muscle inflammation, thereby compounding muscle deterioration and inciting systemic inflammation [45]. The interaction between malnutrition and compromised immune function serves to exacerbate the overall health decline observed in patients [46].

Malnutrition and immune status, as indicated by SII and GNRI, have been well-established as reliable predictors of mortality among elderly patients undergoing hip fracture surgery [22, 27], However, comprehensive assessments of patients’ physical conditions are lacking in the existing literature. Our study offers a novel approach by combining nutritional and immune function factors to create the SII–GNRI score as a new scoring system for predicting the association between poor prognosis and mortality following fracture surgery. As anticipated, our initial findings reveal that patients who did not survive had higher SII–GNRI scores compared to those who did. In the COX regression unifactor analysis, several factors were significantly associated with an increased one-year mortality rate, including older age (≥ 80 years), CCI > 2, fracture site, heart disease, brain disease, anemia, length of hospital stay, blood transfusion, ASA > 2, GNRI ≤ 579.39, SII ≥ 1621.92, low albumin levels, low body weight, and high SII–GNRI score (2–3 points). Upon adjusting for other factors in the multivariate Cox proportional risk model analysis, it was revealed that length of hospital stay, CCI > 2, and blood transfusion were the identified risk factors for one-year mortality after surgery in elderly patients with hip fractures (*P* < 0.05). In particular, high SII–GNRI scores (2–3) stand out as an independent prognostic factor. A SII–GNRI score of 2 (indicating either a high SII with SII ≥ 1621.92 or a low GNRI with GNRI ≤ 579.39) yielded a corrected hazard ratio (HR) of 3.68 (95% CI 1.83–7.40), while a SII–GNRI score of 3 (representing both a high SII with SII ≥ 1621.92 and a low GNRI with GNRI ≤ 579.39) resulted in a calibrated hazard ratio of 8.47 (95% CI 4.10–17.49). These findings underscore the significance of high SII–GNRI scores as an independent predictor.

Evidence from previous studies has consistently shown a close relationship between nutritional status, as indicated by GNRI, and systemic immunoinflammatory factors, as represented by the SII, and postoperative complications (POCs), including conditions such as deep vein thrombosis, pneumonia, and postoperative delirium issues [47–49]. Our own investigation revealed that 98 patients experienced POCs after surgery, with a notably higher incidence of POCs observed in patients with elevated SII–GNRI scores (Table 3; *p* = 0.001). Consequently, our study advocates for the preoperative evaluation of nutritional status and inflammatory indices. Patients with a high SII combined with a low GNRI are projected to have a poor survival rate, emphasizing the importance of promptly implementing appropriate preventive measures for this patient population to reduce mortality and enhance postoperative quality of life.

Low GNRI and high SII levels have been associated with poor outcomes in patients undergoing hip fracture surgery, although the precise underlying mechanism remains unclear and likely involves several contributing factors. Firstly, elderly patients are particularly susceptible to malnutrition due to alterations in their dietary habits. Moreover, their anabolic response to nutritional support is diminished. Anabolic resistance refers to the phenomenon where protein synthesis in muscle tissue is impaired, making it unable to respond adequately to the intake of amino acids and other nutrients. This resistance can be attributed to various factors, including the effects of surgery, trauma, chronic debilitating diseases, aging, and more [50]. Low serum albumin levels are known to be linked with increased levels of inflammatory cytokines such as interleukin-1, interleukin-6, tumor necrosis factor-α, and CRP [51]. This cascade of inflammatory cytokines and chemokines exerts a profound catabolic influence on the patient's metabolism. Furthermore, the release of mitochondrial DNA (mtDNA) into the bloodstream, induced by hip fracture, triggers a systemic inflammatory response and organ damage. This is primarily mediated through the activation of the TLR9/NF-KB pathway [52, 53]. Notably, intramedullary nailing surgery intensifies the rapid release of mtDNA, exacerbating organ damage in hip fracture patients and thereby increasing the risk of mortality [54].

The study presents some limitations. Firstly, it adopted a retrospective design and encompassed patients from a single institution, which rendered it unable to fully address potential bias inherent in observational studies. Secondly, the utilization of laboratory data exclusively at the time of admission to the hip fracture database constrained our capacity to investigate the correlation between SII–GNRI and survival following hip fractures at different time intervals. Thirdly, the study solely comprised patients who underwent surgical treatment and did not assess those undergoing conservative treatment, consequently diminishing its predictive efficacy. Finally, for patients failing to seek timely medical attention and undergo surgery due to trauma, or those unable to receive surgical intervention at the optimal time for medical reasons, adverse effects on their postoperative prognosis may ensue.

## Conclusion

The findings of this study reveal the importance of the combined score of Systemic Inflammation Index and Geriatric Nutritional Risk Index (SII–GNRI), particularly when SII ≥ 1621.92 and GNRI ≤ 579.39. This score emerges as a powerful tool for predicting one-year survival outcomes in elderly patients undergoing hip fracture surgery. It holds potential value in preoperative patient assessment and in making treatment decisions for elderly hip fracture patients.

## Data Availability

The datasets generated during and/or analyzed during the current study are available from the corresponding author on reasonable request.
